# Metal-Modified Montmorillonite as Plasmonic Microstructure for Direct Protein Detection

**DOI:** 10.3390/s21082655

**Published:** 2021-04-09

**Authors:** Giorgia Giovannini, Denis Garoli, Patrick Rupper, Antonia Neels, René M. Rossi, Luciano F. Boesel

**Affiliations:** 1 Empa, Swiss Federal Laboratories for Materials Science and Technology, Laboratory for Biomimetic Membranes and Textiles, Lerchenfeldstrasse 5, CH-9014 St. Gallen, Switzerland; Rene.Rossi@empa.ch; 2 Istituto Italiano di Tecnologia, via Morego 30, 16163 Genova, Italy; denis.garoli@iit.it; 3 Faculty of Science and Technology, Free University of Bozen-Bolzano, Piazza Università, 39100 Bolzano, Italy; 4 Empa, Swiss Federal Laboratories for Materials Science and Technology, Laboratory for Advanced Fibers, Lerchenfeldstrasse 5, CH-9014 St. Gallen, Switzerland; Patrick.Rupper@empa.ch; 5 Empa, Swiss Federal Laboratories for Materials Science and Technology, Center for X-ray Analytics, Lerchenfeldstrasse 5, CH-9014 St. Gallen, Switzerland; Antonia.Neels@empa.ch

**Keywords:** plasmonic, fluorescence, detection, ultraviolet, clay, bio-sensing, SERS, MEF

## Abstract

Thanks to its negative surface charge and high swelling behavior, montmorillonite (MMT) has been widely used to design hybrid materials for applications in metal ion adsorption, drug delivery, or antibacterial substrates. The changes in photophysical and photochemical properties observed when fluorophores interact with MMT make these hybrid materials attractive for designing novel optical sensors. Sensor technology is making huge strides forward, achieving high sensitivity and selectivity, but the fabrication of the sensing platform is often time-consuming and requires expensive chemicals and facilities. Here, we synthesized metal-modified MMT particles suitable for the bio-sensing of self-fluorescent biomolecules. The fluorescent enhancement achieved by combining clay minerals and plasmonic effect was exploited to improve the sensitivity of the fluorescence-based detection mechanism. As proof of concept, we showed that the signal of fluorescein isothiocyanate can be harvested by a factor of 60 using silver-modified MMT, while bovine serum albumin was successfully detected at 1.9 µg/mL. Furthermore, we demonstrated the versatility of the proposed hybrid materials by exploiting their plasmonic properties to develop liquid label-free detection systems. Our results on the signal enhancement achieved using metal-modified MMT will allow the development of highly sensitive, easily fabricated, and cost-efficient fluorescent- and plasmonic-based detection methods for biomolecules.

## 1. Introduction

The Clay is a diverse and abundant class of minerals, peculiar due to the layered structure. The classification of clay minerals is based on their structure, shape, surface charge, presence of interlayered cations and their exchangeability. Clay materials are thus divided into different groups (kaolinite, vermiculite, smectite, illite and chlorite); which have been widely studied in the last decades and have been proposed for different applications spanning from food packaging, drilling fluids and pharmacological formulations [1,2,3]. Montmorillonite (MMT) belongs to the smectite group. It is composed of repeating tetrahedral (Si-O) and octahedral (Al-O) aluminosilicate sheets that bind together by sharing oxygens. Among the members of the smectite group, MMT has the highest swelling capacity in water and therefore shows a good rate of cation exchange, mainly Mg^2+^, Ca^2+^, Fe^2+^, Na^+^ and H^+^ [4] which has been exploited for the synthesis of hybrid materials [5]. Metal-modified MMT hybrid materials have been investigated and proposed for the absorption of metal ions or as substrates showing antibacterial properties [6,7,8]. The changes in the optical properties of dyes upon interaction with MMT are well documented in other papers. The fluorescent enhancement observed with MMT has been mainly associated with the restriction of intermolecular rotation (RIR) and aggregation-induced emission (AIE) phenomenon which can occur after dye-particle interaction [9]. However, the mechanism of signal harvesting varies depending on the optical and physical properties of the molecules studied as we discussed in our previous work. Taking advantage of the remarkable fluorescent enhancement achieved after dye-MMT interaction, we recently proposed the use of clay-organic hybrids for the development of fluorescence-based platforms for sensing biological compounds [9].

A wide variety of fluorescent-based detection approaches has been developed in the last decade. The main strategies are based on: (i) selective labeling of the target with fluorescent nanomaterials or fluorophores [10]; (ii) Föster Resonance Energy Transfer (FRET) or switchable systems that were the fluorescent signal changes upon interaction with the target [11,12]. Despite the promising results, fluorescence-based detection approaches suffer from a few limitations such as low sensitivity and interferences with the background. To overcome such issues, the analyte can be isolated and concentrated or the fluorescent signal can be enhanced improving the signal-to-noise ratio, ultimately improving the limit of detection (LOD) [13]. For instance, metal-enhanced fluorescent (MEF) approaches have been widely explored to increase the sensitivity of detection techniques exploiting the field enhancement in close proximity to metal nanoparticles [14]. The MEF phenomenon is related to the localized surface plasmon resonance (LSPRs) at the particle surface which modifies the optical environment that the fluorophore perceives [15]. Moreover, plasmon-enhancing nanostructures found application in the Surface-Enhanced Raman Scattering (SERS), pushing this labeled-free detection approach to a single-molecule detection [16,17]. Gold and silver have been widely investigated for the development of plasmonic particles for optical applications. However, their use tends to be costly and limited by the relatively low elemental abundance of such precious materials. Aluminum has emerged as an inexpensive, earth-abundant alternative to noble metals for the fabrication of plasmonic material [18]. Even though these strategies allow improving the sensitivity of fluorescence-based detection approaches, the preparation of suitable nanostructures is often laborious, costly and based on different steps.

In the present work, we improved the fluorescent enhancement property of MMT combining it with MEF. The so achieved suspensions of metal-modified MMT are proposed for the design of fluorescence-based sensing and biosensing methods. In particular, MMTs were modified exploiting their high cation exchange capacity for the absorption of silver and aluminum. The hybrid materials allow a remarkable fluorescence enhancement thanks to the combination of clay minerals with plasmonic nanostructures proving to be a valuable alternative to more advanced plasmonic nanostructures which fabrication is time-consuming and requires expensive technologies. Furthermore, Ag-modified MMT was used to enhance the Raman signal exploiting the plasmonic effect of metallic silver, demonstrating the suitability for label-free sensing as well as the fluorescence-based method. Promising results were achieved with these easily prepared and cost-efficient microstructures in detecting self-fluorescent molecules such as fluorescein isothiocyanate (FITC), tryptophan (Trp) and bovine serum albumin (BSA) at low concentration. Thanks to their high detection sensitivity and versatility, being suitable for either fluorescence-based and label-free detection approaches, metal-modified MMT are promising hybrid materials for the further development of highly efficient and affordable detection systems.

## 2. Materials and Methods

### 2.1. Materials

L-tryptophan (Trp) and sodium borohydride (NaBH4) were purchased from Fluka. Fluorescein isothiocyanate (FITC), silver nitrate (AgNO_3_), aluminum chloride (AlCl_3_) and bovine serum albumin (BSA) were purchased from Sigma-Alrich. Sodium montmorillonite (MMT) Dellite^®^LVF was donated by Laviosa Chimica Materia (Livorno, Italy).

### 2.2. Methods

Synthesis of metal-modified MMT:

MMT was dispersed in DI water at the concentration of 0.2 wt% and stirred for three days at R.T. The suspension was sonicated (for 30 min, power 25%, frequency 35 kHz) and centrifuged (at 5765 G-force for 10 min) three times. The suspension of MMT was then stirred with a 0.2 M solution of AgNO_3_ or AlCl_3_ overnight in the volume ratio of 1:1 reaching the final concentration of 0.1 wt% MMT and 0.1 M silver/aluminum salt. A solution of 0.1 M of NaBH4 was added to the reaction to reduce the cation to the metal form and the reaction was stirred overnight. The suspension was dialyzed against DI water (500 mL, changed every 12 h) for three days to remove unreacted chemicals and finally centrifuged (3698 G-force, 8 min) to remove metal nanoparticles not adsorbed on clay particles. For the preparation of pure Ag and Al, the same procedure was followed simply using DI water instead of the suspension of MMT and avoiding the centrifugation step during purification. In the following, the MMT modified with Ag and Al are labeled as Ag_MMT and Al_MMT, respectively.

Transmission electron microscopy (TEM):

Images were taken on a JEOL 2200FS TEM equipped with an in-column Omega-type energy filter (Joel). In total, 5 μL of the sample was added on ‘Carbon Films on 200 Mesh Grids Copper’ and allowed to evaporate.

Scanning electron microscopy (SEM):

SEM images were acquired using a Hitachi S-4800 scanning electron microscope at an acceleration voltage of 20 kV and magnifications from 10 k to 20 k. The samples were fixed on conductive carbon tape and sputter-coated with 7 nm of Au/Pd alloy to facilitate imaging by compensating extensive charging.

Energy-dispersive X-ray (EDX) analysis:

EDX analysis was performed using the above-mentioned SEM equipped with an INCA X-Sight detector (Oxford Instruments). Three independent specimens were measured and an average value was obtained from the resulting elemental compositions.

Surface Enhanced Raman Spectroscopy (SERS):

Renishaw InVia Microscope Raman system with a 50 × 0.95 NA objective was used to perform Raman spectroscopy. The spectra were collected using the following parameters: excitation wavelength of 532 nm; spectral resolution of 2.5 cm^−1^; integration time of 1 s. The intensity of the standard peak at 520 cm^−1^ of silicon substrate was used to calibrate the system.

X-ray photoelectron spectroscopy (XPS):

A VersaProbe II spectrometer (Physical Electronics, Minnesota, USA) with monochromatic Al Kα radiation (1486.6 eV), photoemission take-off angle of 45° (with respect to the surface plane) and operating pressure of the chamber <1 × 10^−6^ Pa was used for XPS measurements. The following measurement parameters were used: (i) survey scan spectra (0–1100 eV): energy step of 0.8 eV; acquisition time of 160 ms per data point; analyzer pass energy of 187.85 eV. (ii) Higher-resolution detail spectra of carbon (C 1s, 278–298 eV), oxygen (O 1s, 523–543 eV), silicon (Si 2p, 94–114 eV), aluminum (Al 2p, 68–88 eV) and silver (Ag 3d, 362–382 eV): energy step of 0.125 eV; acquisition times in between 1.4 s and 4.3 s per data point; analyzer pass energy of 29.35 eV. The energy resolution (full width at half-maximum height) measured on the silver Ag 3d_5/2_ photoemission line is 2.2 eV (for a pass energy of 187.85 eV) and 0.7 eV (for a pass energy of 29.35 eV). All samples (MMT, metal-modified MMT and the reference nanoparticles Al and Ag) were fixed on a stainless steel holder using a double-sided adhesive tape and the sample charging was compensated using dual beam charge neutralization with a flux of low energy electrons (1.5 eV) combined with very low energy positive Ar ions (10 eV). A micro-focused X-ray beam of 100 µm diameter (operated at a power of 25 W at 15 kV) was used to analyze the samples in random positions. The 180° spherical capacitor energy analyzer was operated in the fixed analyzer transmission mode. Obtained spectra were shifted relative to the C-C bond from adventitious hydrocarbon contamination at 285.0 eV. CasaXPS software version 2.3.16 (Casa Software Ltd., Teignmouth, UK) was used to analyze the spectra and curve fitting was carried out using a fixed 70% Gaussian—30% Lorentzian product function. Atomic concentrations were calculated from XPS peak areas after subtracting a Shirley-type background, and tabulated PHI sensitivity factors [19] have been used for quantification. The factors were corrected for our system’s transmission function and spectrometer geometry. Relative uncertainties in the measured concentration are estimated to be approximately ± 10% including uncertainties in the background determination from the energy window setting and transmission function correction.

X-ray diffraction (XRD):

X-ray diffraction patterns of the suspensions have been measured on a PANAlytical MPD Powder (Malvern Panalytical, Almelo, NL, USA) instrument using a Bragg-Brentano setup. Data collection was performed at room temperature using Cu-Kα radiation (λ = 1.5406 Å). After drying of the suspensions, the solids have been mounted on a STOE IPDS-II (Stoe & Cie, Darmstadt, D) instrument equipped with an imaging plate using Mo-Kα radiation (λ = 0.71073 Å) and measured in transmission mode using an X-ray beam diameter of 0.5 mm. Two-dimensional (2D) diffraction images are collected. A 360° integration results in one-dimensional (1D) profiles which have been used for indexing with the program HighScorePlus [20] and the COD [21].

UV-Vis measurements:

UV-Vis spectra of MMT, Ag_MMT, Al_MMT, Ag and Al were recorded diluting the samples 10 times in DI water. A total of 500 μL of the sample was added in a quartz cuvette (500 μL, length path 0.1 cm) and the spectrum was recorded. The spectra of Ag_MMT and Al_MMT are normalized to the spectra of MMT. All reported spectra were acquired using Varian Cary 50Bio connected to 50 MPR (Agilent).

Fluorescent measurements:

In total, 100 μL of the tested samples (MMT, Ag_MMT, Al_MMT, Ag and Al) were mixed in a 96-well plate with 100 μL solution of the targeted analyte (FITC, Trp and BSA) reaching the desired concentration in the final volume of 200 μL (i.e., 10–0.01 nM for FITC; 100–12.5 μM for Trp and 100–25 μg/mL for BSA). The spectra were recorded after 15 min of shaking at room temperature. The excitation and emission wavelength used for Trp and BSA were 260 nm and 360 nm, respectively, while in the case of FITC, specimens were excited at 485 nm and the emission was measured at 520 nm. Spectra were recorded using Varian Cary Eclipse (Agilent).

## 3. Results and Discussion

### 3.1. Synthesis and Characterization of Metal-Modified MMT

**Figure 1:**
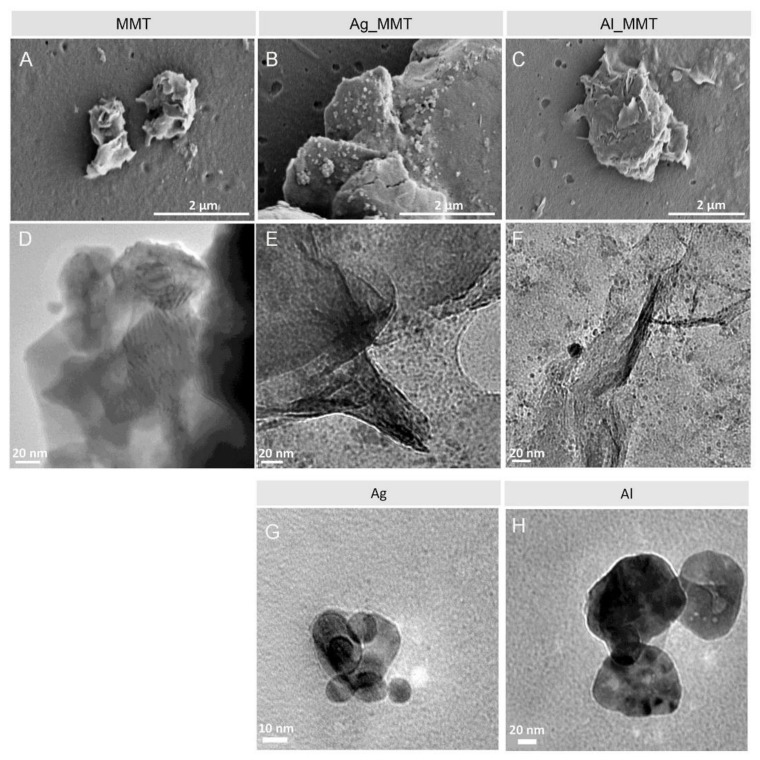
SEM (**A**, **B**, **C**) and TEM (**D**, **E**, **F**) micrographs of pristine clay (MMT), silver (Ag_MMT), aluminum (Al_MMT) modified clay and of Ag and Al nanoparticles (**G** and **H**).

Hydrated MMT particles were modified by absorption of Ag^+^ and Al^3+^ cations, added, respectively, as AgNO_3_ and AlCl_3_ using 0.1 wt% of clay and 0.1 M of cation, and a subsequent reduction with NaBH_4_. The synthesized hybrid clay particles were purified by dialysis and centrifugation and were finally dispersed in DI water. The results achieved with Ag_MMT and Al_MMT were compared with the ones obtained with the pristine MMT and Ag/Al particles that were synthesized following the same procedure mentioned above but without adding MMT. Figure 1 shows electron microscopy images of the materials studied showing the morphology of MMT before and after metal-modification and proving the effective formation of Ag and Al nanoparticles.

**Figure 2:**
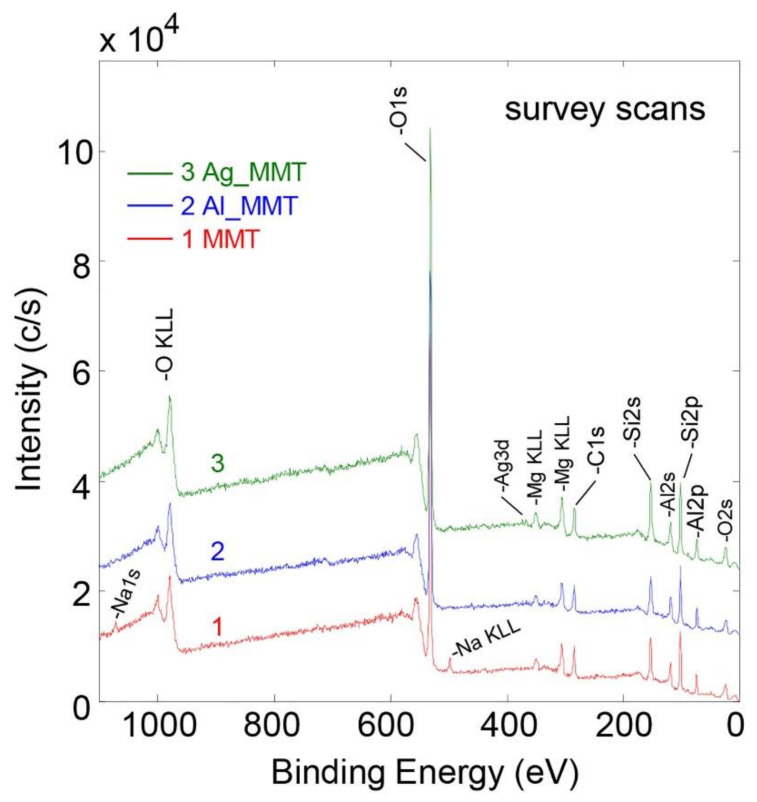
Comparison of XPS survey scan spectra for MMT and their modifications (Al_MMT, Ag_MMT). Scans 2 and 3 are offset in the y-direction compared to scan 1 for better visualization.

XPS measurements determined the elemental composition of the synthesized hybrid materials. Figure 2 depicts XPS survey scans for MMT as well as the modified samples with Al and Ag. The corresponding elemental concentrations are listed in Table 1. The elements silicon (Si), oxygen (O), aluminum (Al) and magnesium (Mg), as expected for MMT are observed in Figure 2 [22,23]. In addition, a carbon (C) signal has been detected, resulting from surface contamination (adventitious carbon) in the order of 10 at.%, as commonly observed in XPS. Sodium (Na) is also present and detected in the MMT sample, however, it is replaced by aluminum as well as silver ions after the modification. The Si/Al ratio (see Table 1) is close to the expected theoretical stoichiometric ratio of 2:1 in MMT [22]. MMT modified with silver showed a clear Ag signal, though with a small concentration of 0.1 at.% (and therefore hardly visible at the chosen intensity scale of Figure 2). Ag was not detected in the MMT nor in the Al_MMT samples, hence proving the success of the silver modification of MMT. A slight increase in the aluminum concentration (approximately in the order of the maximum estimated error) was found in the Al_MMT sample compared to pristine MMT. Therefore, both the Ag_MMT and Al_MMT modifications only introduced a small amount of Ag and additional Al into the MMT, respectively.

**Figure 3:**
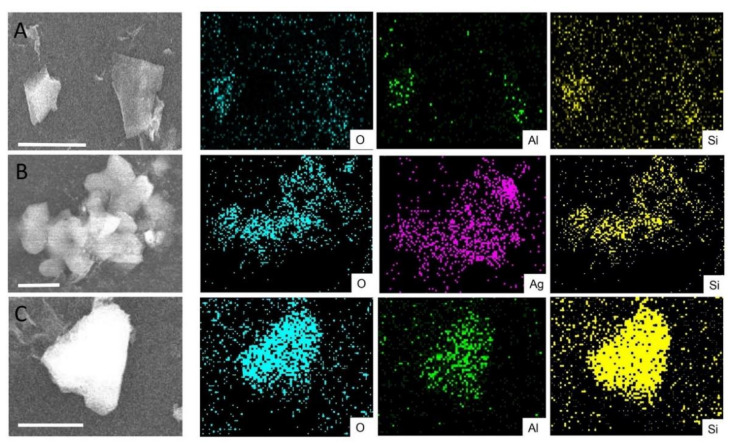
Energy-dispersive X-ray (EDX) mapping analysis of MMT (**A**), Ag_MMT (**B**) and Al_MMT (**C**). The reported scale bars correspond to 2.5 µm.

The lateral distribution of the individual elements was investigated with EDX mapping analysis (Figure 3). Silver was only detected in Ag_MMT and not in the pristine MMT, thereby confirming the results from XPS. Moreover, the Ag is rather homogeneously distributed along with the MMT particles (Figure 3B). For Al_MMT, on average, a higher aluminum concentration was found compared to the unmodified MMT (Figure 3C). However, the values are within the error from different measurements, confirming the results from XPS, where no enhancement of the Al signal was observed for the Al_MMT sample.

**Figure 4:**
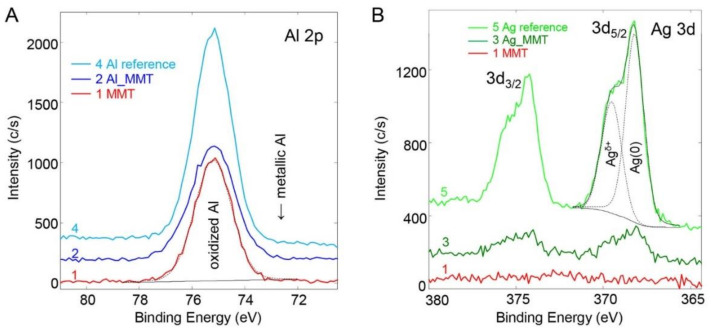
Comparison of XPS spectra for MMT, their modifications (Al_MMT, Ag_MMT) and reference nanoparticle samples Al and Ag. High-resolution elemental scans for aluminum Al 2p (**A**) and silver Ag 3d (**B**). Full lines represent the experimental spectrum, the dotted lines are the bands from the curve fitting into the different functional components. The spectra are offset in the y-direction for better visualization.

XPS analysis further allowed the investigation of Ag and Al electronic states. Figure 4A,B depict the corresponding high-resolution scans for Al 2p and Ag 3d. Aluminum bonds involving oxygen (oxides Al_2_O_3_; hydroxides AlOOH, Al(OH)_3_) and aluminum ions present in alumino-silicate minerals all possess similar binding energies for Al 2p between 74.5 eV and 76 eV [24,25,26,27]. Therefore, they cannot be clearly resolved and have been fitted together as one component. On the other hand, metallic aluminum Al(0) would be significantly shifted (>2 eV) towards lower binding energies. As observed from Figure 4A, the Al 2p spectrum shows no evidence of a metallic contribution. This prevalence of aluminum in its oxidized state can be emphasized by the fact that the hybrid material was synthesized in water. From the XPS data, the amount of aluminum additionally present in the modified MMT sample is low compared to aluminum already present in MMT (see Table 1). Therefore, it is possible that part of the additional Al present in Al_MMT is nevertheless in the metallic form, the signal of which would be buried in the noise of the XPS spectrum.

The Ag 3d spectra are shown in Figure 4B. As already observed in the survey scans, no silver was present in the MMT sample. The Ag concentration in the Ag_MMT sample is low (0.1 at.%, i.e., at the detection limit of XPS under our conditions). However, the shape and energetic position of the peaks are very similar to the one from the reference Ag nanoparticle sample (with an increased silver concentration), which is also shown in Figure 4B. The maxima of the two bands at 368.1 eV and 374.2 eV correspond to the binding energies of the two spin-orbit doublets of Ag 3d_5/2_ and Ag 3d_3/2_, respectively. The energetic positions, as well as the splitting, are in good agreement with the well-known literature data for metallic silver Ag(0) [28,29,30]. No bands were observed corresponding to oxide species Ag(I/II), which are located between 367 eV and 368 eV [29,31]. While metallic silver is assigned as the major component of the peak, a broader shoulder towards higher binding energy around 369.4 eV is observed in Figure 4B. We assigned this band to metallic silver with a slight increase in oxidation state (Ag^δ+^) arising from the interaction of the metallic silver nanoparticles and its MMT support, and hence, leading to an increase in the binding energy of metallic Ag. Such a mechanism is described in the literature [28,32,33]. In summary, for Ag_MMT silver is mostly present in the form of metal, whereas, for Al_MMT, the majority of the additional aluminum is oxidized.

**Figure 5:**
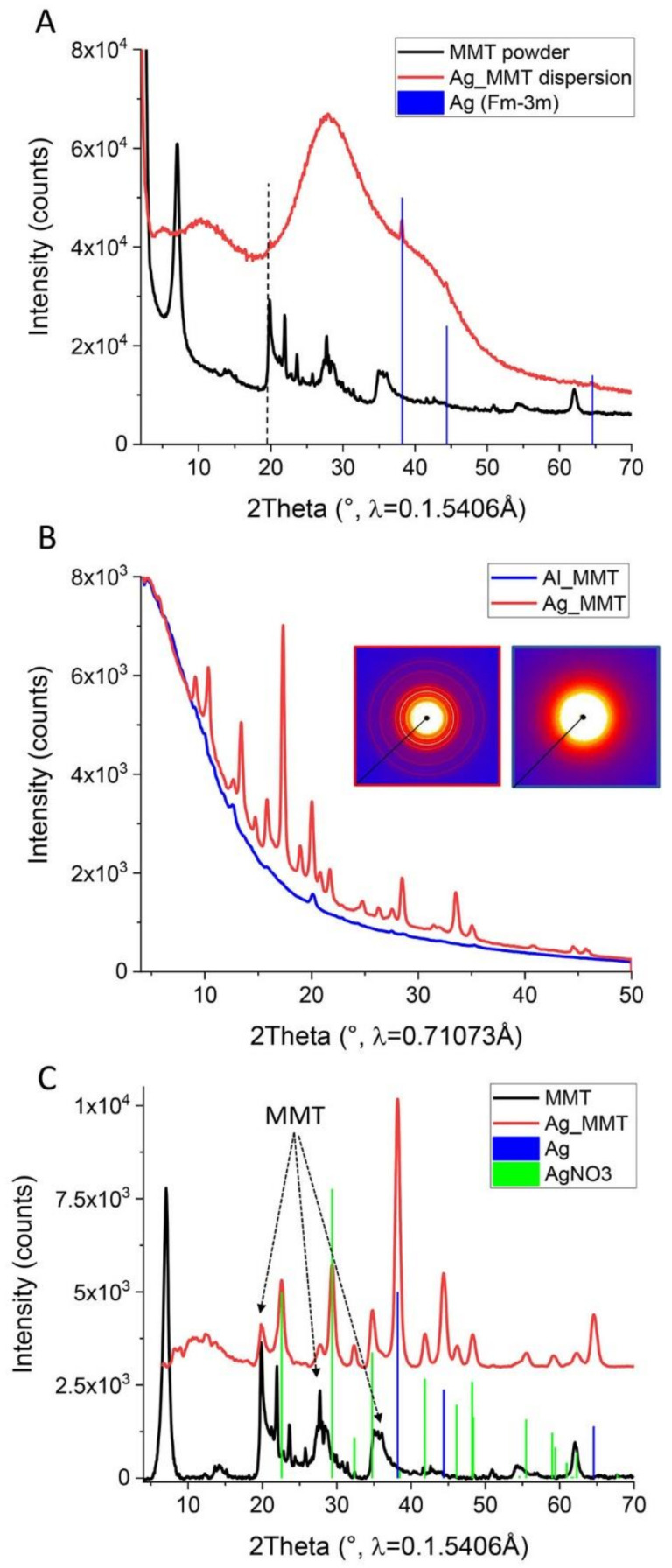
(**A**) XRD pattern of Ag_MMT prepared as water suspensions and MMT powder as a control. Assignment of peaks of metallic silver in Ag_MMT (blue lines: cubic Ag phase, Fm-3 m, with a = 4.079 Å). The small peak at 19.9° (gray dotted line) may refer to the *d*_100_ of MMT. The three very broad hallos centered at 11°, 29°, and 42° are from water (not shown). (**B**) XRD patterns of the solid samples of Ag_MMT (red line) and Al_MMT (blue line). The insets show the original 2D diffraction images, from which the 1D patterns were obtained (left: Ag_MMT; right: Al_MMT). (**C**) Assignment of peaks for metallic silver in Ag_MMT (blue lines: cubic Ag phase with Fm-3 m, a = 4.079 Å), green lines: hexagonal AgNO_3_ phase. A few peaks refer to reflections from the MMT: 20° (*d*_003_ and *d*_100_), 28° (*d*004), 35° (*d*_005_ and *d*_105_). For this graph, the x-axis of the curve of Ag_MMT was converted to represent the use of Cu Ka radiation. For Al_MMT, the signal-to-noise ratio was too small to allow conclusive peak assignment.

X-ray diffraction (XRD) patterns were obtained for samples in dispersion (Figure 5A) and solid form (Figure 5B,C). Due to the high dilution of the samples in the dispersion (only 0.1% of solids in water), most of the features in the curves of MMT, Ag_MMT, and Al_MMT correspond to those of pure water, specifically the three very broad hallos centered at 11°, 29°, and 42° 2Theta. Characteristic peaks of the clay are absent both due to the high dilution (only 0.1% of solids in water) as well as to exfoliation (that is, the disappearance of the strong *d*_001_ reflection). A few weak peaks corresponding to the metallic silver phase (cubic Fm-3 m, a = 4.079 Å) could be observed (see peak assignment in Figure 5A), together with a weak peak attributed to the *d*_100_ reflection of MMT (19.9°). No peaks for metallic aluminum could be found in Al_MMT (data not shown).

2D diffraction images of the solid samples show only for Ag_MMT the presence of rings (crystalline material) superimposed on the amorphous background of the samples (Figure 5B, insets). The peaks for metallic silver could be identified in the 1D diffraction patterns (Figure 5C), confirming their presence on the clay platelets and the hypothesis that silver is mostly found in the metallic form (COD PDF No. 96-901-2432). Silver nitrate (Ag(NO_3_), COD PDF No. 96-210-5350) was also observed, due to its presence in the initial reaction medium. The remaining peaks (marked with arrows in Figure 5C) correspond to the reflections of clay (*d*_100_, *d*_105_, and a few *d*_00*l*_ reflections). It should be noted that some peaks between 22° and 37° 2Theta correspond to contributions of both MMT and Ag(NO_3_) and cannot be uniquely identified. The absence of the strong *d*_001_ reflection of MMT is probably attributed to the sample preparation and related MMT exfoliation; the diffractogram of the powder with stacks of platelets shows this reflection [34]. For Al_MMT, no peaks attributed to the metal could be observed in the X-ray diffraction pattern: instead, only reflections from oxidized Al or the MMT are present as weak peaks. Possible candidates are hydroxides or carbonates such as AlO(OH) or (NH_4_)Al(CO_3_)(OH)_2_ but the signal-to-noise ratio is too small for firm indexing. This corroborates XPS and EDX analyses that the small amount of Al deposited on the platelets is most probably oxidized and therefore difficult to differentiate from the Al already present in the structure of pristine MMT (Table 1 and Figure 4A). In summary, XRD of both dispersions and solid samples agrees with the EDX and XPS analyses: metallic material could only be identified in Ag_MMT (together with a minor second Ag(NO_3_) phase), while oxidized aluminum was present in Al_MMT.

**Figure 6:**
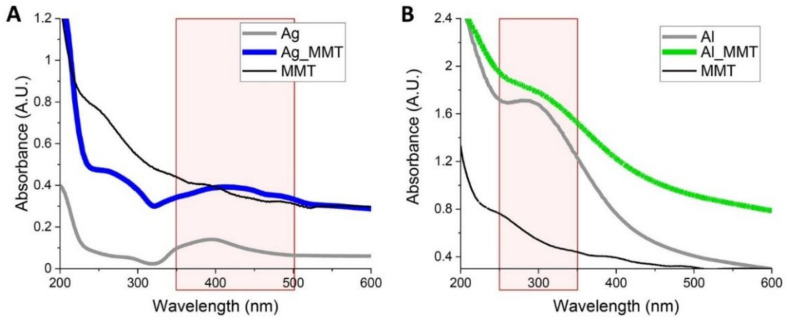
UV-Vis analysis of (**A**) Ag and Ag_MMT showing a broad absorbance peak between 350 and 500 nm and (**B**) Al and Al_MMT which absorb in the deep UV, 250–350 nm. The reference curves of Ag and Al reported in the graphs correspond to the spectra of the synthesized nanoparticles. Spectra of Ag_MMT and Al_MMT were normalized to the spectrum of MMT.

The peaks observed in the UV-Vis absorption spectra at 400 nm for Ag_MMT (Figure 6A) and at 280 nm for Al_MMT (Figure 6B) confirmed the presence of two metals in the MMT. Indeed, Ag nanoparticles are characterized by a broad absorption peak around 400 nm while Al nanoparticles absorb below 300 nm, as confirmed by the spectra recorded for the synthesized Ag and Al nanoparticles (grey lines in Figure 6A,B). This analysis supported the results achieved with XPS, EDX and XRD confirming the successful modification of MMT. The spectra were normalized to the one measured for MMT to remove the strong signal below 300 nm characteristic for Si-based materials. To achieve fluorescent enhancement, the chosen metal and the fluorophore have to absorb in the same range of wavelength. Hence, MMT was modified with Ag to achieve a fluorescent enhancement in the visible range (400–500 nm) [35,36] and with Al allowing the enhancement in the deep-UV (200–300 nm) [37,38]. The difference in the UV spectrum determines the different applications for Ag_MMT and Al_MMT. Indeed, while Al-based materials are suitable for the fluorescence-based detection of biomolecules such as proteins absorbing in the deep-UV, Ag-based structures can be exploited to enhance fluorescent signals of molecules absorbing in the UV-Vis such as FITC and coumarin derivatives for instance.

### 3.2. Fluorescence-Based Detection of Biomolecules with Al_MMT

**Figure 7:**
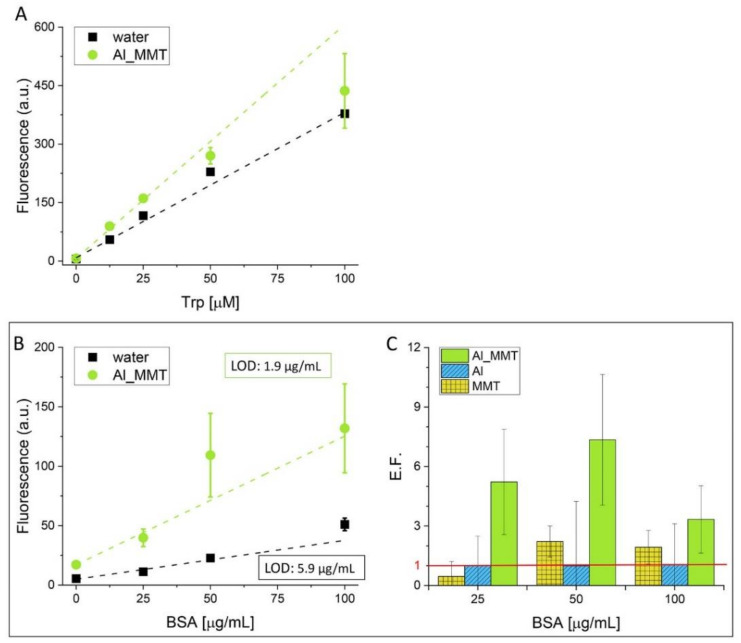
(**A**) Calibration curves obtained by measuring the fluorescent signal of Trp at different concentrations (12.5–100 μM) in water and in Al_MMT suspension (λex 280 nm; λem 360 nm). (**B**) Calibration curves obtained by measuring the fluorescent signal of BSA at different concentrations (25–100 μg/mL) in water and in Al_MMT suspension (λex 280 nm; λem 360 nm). Weighted linear regression fit: (i) water-residual sum of squares 18.1, R-square 0.939; (ii) Al_MMT- residual sum of square 1.56; R-square 0.938. (**C**) enhancement factor (E.F.) calculated for each sample (Al_MMT, MMT, Al) proving the combined effect of MMT and Al in the hybrid material Al_MMT. Values are reported as mean ± sd (n = 3).

First, we evaluated the suitability of Al_MMT in detecting self-fluorescent amino acid tryptophan, enhancing its intrinsic fluorescence [39]. The limit of detection (LOD) calculated (according to the formula LOD= 3.3(sd/slope) [40] where sd is the standard deviation of the blank) for Trp dispersed inAl_MMT is similar to the one calculated in water (0.54 μM and 0.74 μM respectively) but the experiment proved the applicability of the detection mechanism (Figure 7A). Indeed, the direct detection of protein by means of fluorescence-based approaches takes advantage of the presence of the self-fluorescent Trp in the biomolecule’s structure. Bovine serum albumin has been used extensively as a protein model for the development of bioassay due to its high structural affinity to the human serum albumin, whose levels in biological fluids are closely related to the patient’s health [41]. Containing 3 units of Trp, BSA is characterized by self-fluorescence in the UV range and it was chosen to test the efficiency of Al_MMT in enhancing the fluorescent signal of protein. The fluorescence intensity measured for BSA in water solution at different concentrations was compared with the intensity measured treating the protein with the hybrid structure Al_MMT (Figure 7B). The enhancement factor (E.F.) was determined by dividing the intensity of the fluorescent signal measured for the sample tested by the one measured for the amino acid in solution (water). The E.F. achieved with Al_MMT was compared with the one measured for Al and MMT (Figure 7C). While no enhancement was observed with Al particles, the intrinsic protein’s signal was doubled using MMT. On the other hand, using Al_MMT an enhancement factor of 7.3 was achieved for 50 μg/mL of BSA confirming the efficiency of the hybrid material in enhancing the fluorescent signal of biomolecules. The low enhancing efficiency of Al_MMT treated with Trp can be explained by the low absorption of Trp of MMT due to the counterbalance of either positive (-NH_2_) and negative (-COOH) groups in the aminoacidic structure. On the contrary, different studies showed the prompt absorption of BSA at the surface or the edges of MMT by electrostatic and/or chemical interaction [42,43]. Noticeably, while the fluorescent signal measured for Trp was reproducible between different tests (small standard deviation) both in water and with Al_MMT (Figure 7A), the reproducibility between measurements was observed only in water in the case of BSA. We explain the large sd noted for BSA treated with Al_MMT with the folded structure of the proteins. Indeed, not all the Trp units in the protein are available for interacting with the hybrid material since they can be hindered in the internal part of the protein [44]. Hence, the fluorescent signal is not homogeneously amplified. However, the LOD in detecting BSA enhancing its self-fluorescence by means of Al_MMT was calculated as 1.9 μg/mL (28 nM), which is improved compared to the one achieved in water (5.9 μg/mL) and to other fluorescence-based methods found in the literature. For instance, the fisetin-albumin complex allowed the detection of BSA at concentrations above 10 μg/mL [45] while the fluorescent signal generated after interaction between a pyrrole derivative and BSA due to the aggregation-induced emission phenomenon allows detecting the protein with a calculated LOD of 180 nM [46].

### 3.3. Fluorescence-Based Detection of FITC with Ag_MMT

**Figure 8:**
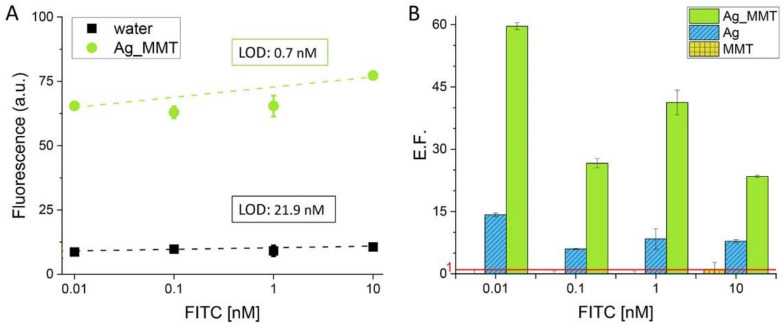
(**A**) Calibration curves obtained by measuring the fluorescent signal of fluorescein isothiocyanate (FITC) at different concentrations (0.01–10 nM) in water and in Ag_MMT suspension (λex 490 nm; λem 520 nm). Weighted linear regression fit: (i) water- residual sum of squares 1.11, R-square 0.802; (ii) Ag_MMT- residual sum of square 10.7; R-square 0.992. (**B**) E.F. calculated for each sample: Ag_MMT, MMT, Ag. Values are reported as mean ± sd (= 3).

Ag_MMT is suitable for the detection of fluorophores which emit in the range of 400–500 nm. As proof of concept, we tested the fluorescent enhancement efficiency of Ag_MMT using FITC, a fluorophore commonly used in designing detection assays and for bio-imaging. The fluorescent enhancement was determined by comparing the values achieved for the dye in an aqueous solution. As shown in Figure 8, Ag_MMT is efficient in increasing the signal of FITC reaching an E.F. of 60 for the lowest concentration tested (0.01 nM). In this study, the effect of combining metallic structures and MMT is particularly evident considering the low enhancement observed with the MMT. As observed in our previous work [9], the fluorescent enhancement achieved using MMT is related to the amount of clay employed as well as to the concentration of the dye. In this work, we used a low concentration of MMT (0.1 wt%) and pico- and nano-molar concentrations of FITC. On the other hand, the photoluminescent harvesting efficacy of Ag nanoparticles (AgNP) is well documented in the literature thanks to their ability to concentrate and amplify light. In particular, the harvesting efficiency of AgNP is higher when the fluorophore is located between coupled metal particles rather than if it interacts with a single particle, due to the higher electric field intensity in-between particles. For instance, Zhang et al. showed that the MEF is almost doubled (when cyanine dye is between coupled AgNP rather than adsorbed on single particles [47]. For this reason, plasmonic metasurfaces have been fabricated in which AgNP are deposited on support films and at a specific distance reaching the coupling [48]. We explained the remarkable E.F. observed using Ag_MMT with the supportive role played by MMT for the adsorbed Ag nanoparticles and the homogeneous distribution of Ag on the MMT surface observed by EDX (Figure 3B). Indeed, the E.F. calculated at different concentrations of FITC for Ag in suspension (E.F. between 6 and 14) are similar to the one found in the literature for AgNP (E.F. 7) while the values observed for Ag_MMT (E.F. between 25–60) are comparable with the one achieved using surfaces coated with AgNP (e.g., 71) [49] with the advance of the easy and cost-efficient fabrication of the metal-modified MMT. Thanks to the signal harvesting, Ag_MMT allowed achieving a 30-times lower LOD compared to the one calculated in water (0.7 nM and 21.9 nM, respectively). This leads to a significant improvement in the sensitivity of fluorescence-based detection approaches, overcoming one of the main limitations of fluorescence-based detection, as discussed previously.

### 3.4. Label-Free Detection: SERS Measurements

Taking into consideration the ability of the hybrid MMT systems to enhance the fluorescence efficiency, we decided to explore potential applications in label-free spectroscopies. Raman and its plasmonic counterpart, SERS, are very powerful tools to measure the spectroscopic fingerprint of resonant molecules [50]. In particular, in SERS, the presence of metallic nanostructures, activating LSPRs in the vicinity of the metallic surface, allows increasing the scattering efficiency by several orders of magnitude [51]. Such improvement enabled achieving single-molecule detection, which represents a hot topic in the field of DNA and protein sequencing when exploited to discriminate between single nucleic acid and amino acid of polymeric chains [16,52,53]. Among others, AgNP provides excellent resonance behaviors for amplifying the signal in the visible range and in comparison to gold nanoparticles, AgNPs allow achieving a stronger plasmonic enhancement due to lower interferences between intraband and interband electronic transition [54].

**Figure 9:**
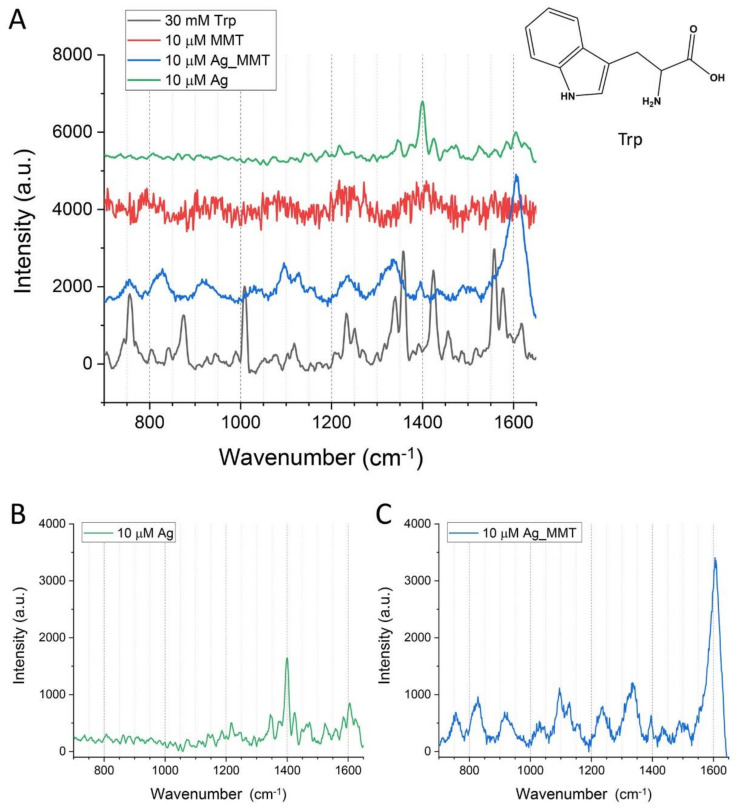
(**A**) Comparison of the SERS spectra of Trp at 30 mM in aqueous solution with the spectrum acquired after incubation of Trp (10 µM) with MMT, Ag_MMT and Ag. The spectra are offset in the y-direction for better visualization. Signal enhancement is achieved using Ag (**B**) and Ag_MMT (**C**). 100 s acquisition time for the aqueous solution and 10 s for the other samples; all the curves are smoothed using a 10-point FFT filter.

To prove the wide versatility and applicability of metal-modified MMT label-free detection of Trp with SERS was conducted. The working range of Ag_MMT is particularly suitable to harvest the Raman signal of Trp using instrumentation commonly available in laboratories. Figure 9A reports the experimental results from all samples (MMT, Ag_MMT and Ag) treated with 10 µM solution of Trp (10 s integration time). The spectra can be compared with Trp Raman spectra from 30 mM aqueous solution since at lower concentrations no signals were detected (100 s integration time).

While MMT alone does not enable achieving a clear Raman spectrum, both Ag and Ag_MMT systems that allow the detection of vibration peaks of Trp. Silver colloidal solutions have been extensively reported as efficient systems in enhancing the SERS spectra of Trp even at nanomolar concentrations [55]. This preliminary result clearly shows that Ag_MMT can perform better than Ag nanoparticles in enhancing the intensity of the Raman signal as shown in Figure 9B,C. A discussion of the Raman shift identification is out of the scope of the present work, but it is possible to identify all the major peaks observed in the literature [56]. Interestingly, the strong Raman shift at 1600 cm^−1^ (assigned to the phenyl ring CC stretch) [57] observed treating Trp with Ag_MMT suggests that the interaction between the amino acid and the hybrid material occurs through the indole ring while the spectrum of Trp treated with Ag indicates that the molecules interact with the metal nanoparticles surface through the carboxyl and amino groups [57]. In view of our results and of the above considerations, we confirmed the hypothesis that MMT act as support for the metallic material rather than being directly involved in the enhancement of the SERS signals which can be due to the favorable and homogeneous deposition of Ag nanoparticles within the clay (as shown in Figure 3B). Moreover, the MMT could improve both frequency and efficiency of molecular bonding, ultimately improving the sensitivity of the detection. These results prove the potential of such easily synthesized and cost-efficient hybrid materials in improving label-free detection methods based on SERS.

## 4. Conclusions

In this work, we present cost-efficient and easily synthesized nanomaterials based on Ag and Al modified-MMT. The achieved hybrid materials showed promising properties in enhancing the fluorescent signal in different spectral regions. In particular, Al_MMT can enhance the signal of a self-fluorescent biomolecule such as proteins that absorb in the deep-UV (200–300 nm) allowing their direct detection without the need to engage labeled-antibody or other expedients, whereas Ag_MMT can enhance the signal of fluorophores such as FITC having absorbance peaks in the visible range and could be applied to improve the LOD of the fluorescence-based sandwich assay (i.e., ELISA tests). The combined effect between MMT and the metallic moiety, which is ascribed to the AIE and plasmonic phenomena, leads to a remarkable fluorescent enhancement that can be used to improve the sensitivity of fluorescence-based detection mechanisms. To demonstrate the versatility of these hybrid materials, Ag_MMT was further tested in enhancing the Raman signal of Trp. The results showed that such metal clay particles are suitable for the development of highly sensitive fluorescence-based and label-free detection systems of (bio-)molecules.

## Figures and Tables

**Table 1:** XPS analysis of MMT and their modifications. Elemental compositions are determined from survey scans. The values are reported as atomic percentage concentrations (at.%) and have been normalized to 100%.

Sample	Si	O	Al	Mg	Na	Ag	C
MMT	15.4	60.3	7.2	1.5	2.0	-	13.6
Ag_MMT	16.2	63.0	7.0	1.8	-	0.1	11.9
Al_MMT	15.6	62.9	7.7	1.9	-	-	11.9

## Data Availability

Data available in a publicly accessible repository. The data presented in this study are openly available in Zenodo at doi:10.5281/zenodo.4593041.
